# Transactivation of TrkB by Sigma-1 receptor mediates cocaine-induced changes in dendritic spine density and morphology in hippocampal and cortical neurons

**DOI:** 10.1038/cddis.2016.319

**Published:** 2016-10-13

**Authors:** Minhan Ka, Yeon-Hee Kook, Ke Liao, Shilpa Buch, Woo-Yang Kim

**Affiliations:** 1 Department of Developmental Neuroscience, Munroe-Meyer Institute, University of Nebraska Medical Center, Omaha, NE 68198, USA; 2 Department of Pharmacology and Experimental Neuroscience, University of Nebraska Medical Center, Omaha, NE 68198, USA

## Abstract

Cocaine is a highly addictive narcotic associated with dendritic spine plasticity in the striatum. However, it remains elusive whether cocaine modifies spines in a cell type-specific or region-specific manner or whether it alters different types of synapses in the brain. In addition, there is a paucity of data on the regulatory mechanism(s) involved in cocaine-induced modification of spine density. In the current study, we report that cocaine exposure differentially alters spine density, spine morphology, and the types of synapses in hippocampal and cortical neurons. Cocaine exposure in the hippocampus resulted in increased spine density, but had no significant effect on cortical neurons. Although cocaine exposure altered spine morphology in both cell types, the patterns of spine morphology were distinct for each cell type. Furthermore, we observed that cocaine selectively affects the density of excitatory synapses. Intriguingly, in hippocampal neurons cocaine-mediated effects on spine density and morphology involved sigma-1 receptor (Sig-1 R) and its downstream TrkB signaling, which were not the case in cortical neurons. Furthermore, pharmacological inhibition of Sig-1 R prevented cocaine-induced TrkB activation in hippocampal neurons. Our findings reveal a novel mechanism by which cocaine induces selective changes in spine morphology, spine density, and synapse formation, and could provide insights into the cellular basis for the cognitive impairment observed in cocaine addicts.

Exposure to cocaine has been shown to interrupt normal cognition and memory, leading to brain malfunction and addiction.^1, 2^ Most studies involving cocaine administration have focused on the nucleus accumbens (NA), an area of the brain in the ventral striatum that receives rich dopaminergic innervation from the ventral tegmental area (VTA).^3, 4^ This neural circuit is central to transmitting the reward sensations related to drug addiction.^5, 6^ However, there are other brain regions in this circuit, such as the hippocampus and cerebral cortex, that are known to participate in associative processes including learning and memory.^7, 8^ The hippocampus is directly connected to the NA and VTA, and can contribute to the activity of dopaminergic neurons in the VTA.^9, 10^ Neurons in the cerebral cortex also have a role in dopamine reward circuitry.^11, 12^

The dendritic spine is the major site of synapse formation in neurons. The number and morphology of dendritic spines can be adjusted in response to activity and experience.^13^ Formation of new dendritic spines and elimination or structural modification of existing spines have been proposed as mechanisms of synaptic plasticity, which in turn is involved in learning, memory, and addiction.^14, 15^ Cocaine administration in rats causes learning and memory deficits,^16, 17^ suggesting that cocaine could elicit structural changes in hippocampal and/or cortical spines. The molecular mechanism(s) of cocaine-mediated effects on spine density and synapse alteration, however, remain only partially understood.

sigma-1 receptor (Sig-1 R) is a brain-enriched transmembrane protein that interacts with various receptors, including G-protein coupled and receptor tyrosine kinases.^18, 19^ Cocaine is a Sig-1 R agonist.^20^ With the binding of cocaine Sig-1 R is activated and translocated to the plasma membrane, where the receptor interacts with various cellular targets critical for neuropsychiatric diseases.^21^ Brain-derived neurotrophic factor (BDNF) and its receptor, TrkB, have an important role in the formation of neural circuits related to learning and memory. For example, BDNF/TrkB signaling is required for dendritic outgrowth and dendritic spine formation in hippocampal neurons.^22, 23^ The interaction of BDNF with the TrkB receptor activates the Ras/ERK, PLC-γ, and phosphatidylinositol 3-kinase/AKT pathways.^24, 25, 26^ Activation of the PLC-*γ* pathway directly induces a rise in intracellular Ca^2+^ and in the activation of the Ca^2+^/calmodulin-dependent kinase (CaMKII).^27, 28^ BDNF expression is, in part, regulated by a positive feedback mechanism via CaMKII activation of cAMP response element-binding protein (CREB).^1, 29^ Rac small GTP-binding proteins are master regulators of actin cytoskeletons and play important roles in the formation of dendritic spines and synapses.^30, 31, 32, 33, 34^ Interestingly, Rac1 GTPase is a target of Sig-1 R and mediates dendritic spine formation in hippocampal neurons.^35^

Herein we report that cocaine differentially regulates the number and morphology of dendritic spines in hippocampal and cortical neurons. Cocaine also modifies the balance of excitatory and inhibitory synapses. In addition, transactivation of TrkB signaling by Sig-1 R mediates cocaine-induced spine changes. Overall, our findings describe a cell type-specific effect and a new mechanism for cocaine-induced spine plasticity.

## Results

### Cocaine differentially regulates dendritic spine density and morphology in cultured hippocampal and cortical neurons

**Figure 1:**
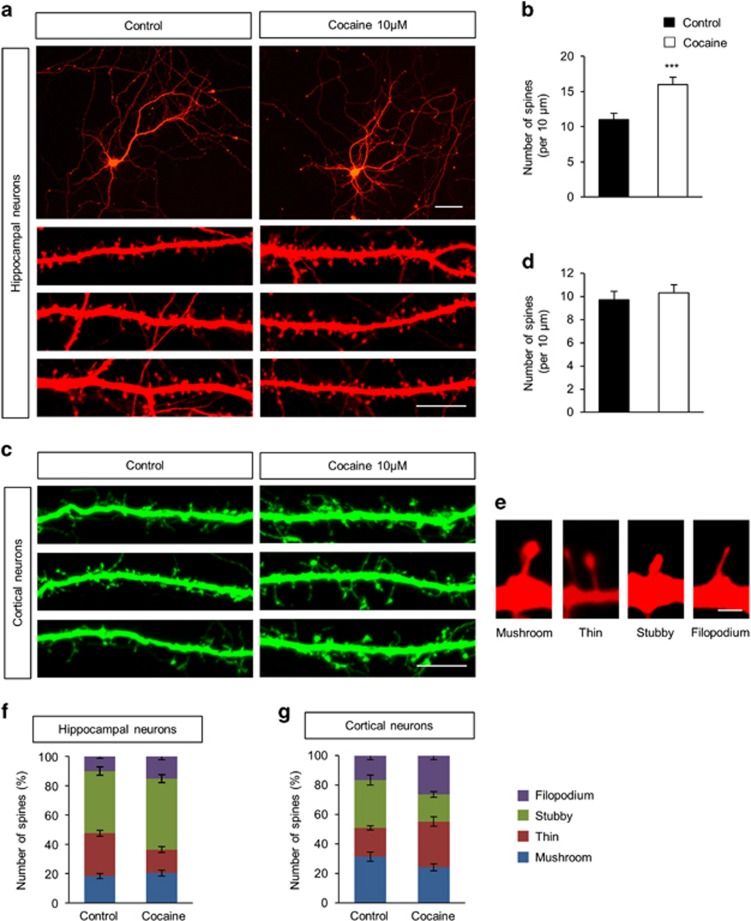
Cocaine differentially regulates dendritic spines in cultured hippocampal and cortical neurons. (**a**) Cocaine modifies dendrites spine density in cultured hippocampal neurons. Primary hippocampal neurons were isolated from E18 embryos, cultured, and transfected with a RFP plasmid at 10 day *in vitro* (DIV). After 6 days, neurons were treated with 10 *μ*M cocaine for 24 h. Scale bar, 25 and 10 *μ*m. (**b**) The number of dendritic spines was quantified. *n*=45 cells from five independent cultures using five rats for each condition. Statistical significance was determined by two-tailed Student's *t*-test. ***P<0.001. (**c**) Cocaine did not alter dendritic spine density in cultured cortical neurons. Scale bar, 10 *μ*m. (**d**) The number of dendritic spines was quantified. *n*=45 cells from five independent cultures using five rats for each condition. (**e**) Different types of dendritic spines (Mushroom, Thin, Stubby, and Filopodium). Scale bar, 1 *μ*m. (**f**), (**g**) Cocaine remodels the composition of spine types in cultured hippocampal and cortical neurons. *n*=45 hippocampal neurons and 992 dendritic spines for control; *n*=45 hippocampal neurons and 1212 dendritic spines for cocaine-treated condition; *n*=45 cortical neurons and 1072 dendritic spines for control; *n*=45 neurons and 1334 dendritic spines for cocaine-treated condition

To examine the effect of cocaine in different cell types, we cultured hippocampal and cortical neurons from embryonic day 18 (E18) rats for 10 days and transfected them with a plasmid encoding either red or green fluorescent protein. After 6 days, neuronal cultures were exposed to either cocaine (10 *μ*M) or saline (control) for 24 h and dendritic spines were assessed using fluorescence microscopy. As shown in Figures 1a and b, exposure of hippocampal neurons to cocaine resulted in increased numbers (~45%) of dendritic spines over control cells. Interestingly, exposure of cortical neurons to cocaine did not alter the number of dendritic spines (Figures 1c and d).

Activity-dependent alteration of spine morphology has an important role in neuronal plasticity.^36, 37^ Thus, we sought to investigate whether cocaine administration could alter the remodeling of dendritic spine morphology. Dendritic spines exhibit varying morphology and can be classified into categories such as filopodia, thin, mushroom, or stubby spines (Figure 1e). Filopodia are typically longer (>2 *μ*m) and normally have no clear head; thin spines have a thin, long neck (>1 *μ*m) and a small heads; mushroom spines have a short and narrow neck (<1 *μ*m) and a large head (>0.6 *μ*m), whereas the stubby spines have a head but no neck. In hippocampal cultures at baseline dendritic spines were comprised of 10% filopodia, 42% stubby, 30% thin, and 18% mushroom spines (Figure 1f). Following cocaine exposure to hippocampal neurons there was an increase in filopodia-like spines by 51% and stubby spines by 15%, compared with controls. In addition, in hippocampal neurons, cocaine exposure also resulted in a significant decrease in the number of thin spines (~45%) compared with neurons not exposed to cocaine. There was no significant change in the number of mushroom spines following cocaine exposure in these cells. In cortical neurons baseline levels of various spines were 32% mushroom, 20% thin, 32% stubby, and 16% filopodia (Figure 1g). Contrary to hippocampal cultures, cortical neurons in the presence of cocaine showed a reduction in stubby spines (~43%) with a concomitant increase in thin (~59%) and filopodia (~59%) spines, compared with control cells. Cocaine thus differentially regulates the number and morphology of dendritic spines in hippocampal and cortical neurons.

### Cocaine induces excitatory synapses in hippocampal neurons

**Figure 2:**
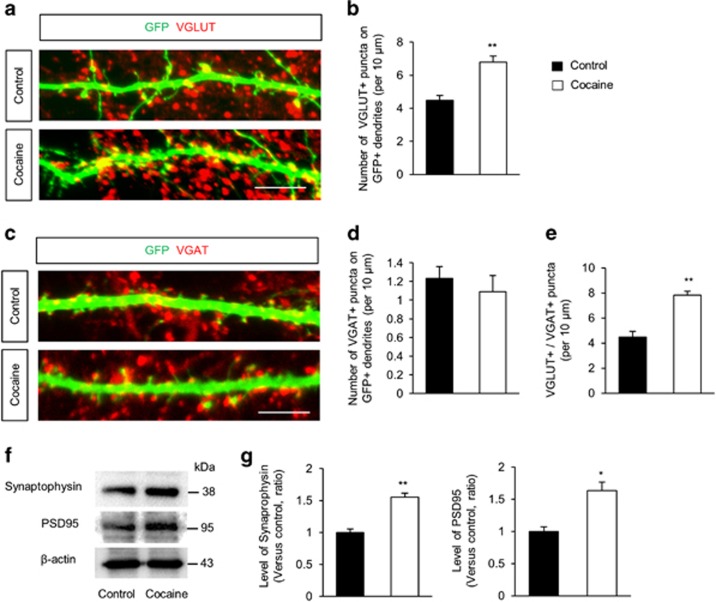
Cocaine increases excitatory synapses in cultured hippocampal neurons. (**a**) Hippocampal neurons from E18 rats were cultured for 10 days and transfected with a GFP plasmid. After 6 days, neurons were treated with 10 *μ*M cocaine for 24 h. Excitatory synapses were assessed by immunostaining using a vGlut1 antibody. Scale bar, 10 *μ*m. (**b**) Quantification of the number of excitatory synapses shown in (**a**). *n*=40 cells from five independent cultures using five rats for each condition. Statistical significance was determined by two-tailed Student's *t*-test. **P<0.01. (**c**) Cocaine induces no changes in the number of inhibitory synapses in cultured hippocampal neurons. Inhibitory synapses were assessed by immunostaining using a vGAT antibody. Scale bar, 10* μ*m. (**d**) Quantification of inhibitory synapse numbers shown in (**c**). *n*=40 cells from five independent cultures using five rats for each condition. Statistical significance was determined by two-tailed Student's *t*-test. (**e**) Cocaine changes the balance of excitatory and inhibitory synaptic puncta. *n*=40 cells from five independent cultures using five rats for each condition. Statistical significance was determined by two-tailed Student's *t*-test. **P<0.01. (**f**) Cocaine increases presynaptic and postsynaptic molecules in hippocampal neurons. Cellular lysates were isolated from neurons treated with 10 *μ*M cocaine for 24 h. Western blotting was performed with a synaptophysin and a PSD95 antibodies. (**g**) Quantification of protein levels shown in (**f**). The levels of protein were normalized to β-actin expression. *n*=3 independent cultures using three rats. Statistical significance was determined by two-tailed Student's *t*-test. *P<0.05, **P<0.01

Having determined that cocaine upregulates dendritic spine density in hippocampal neurons (Figure 1), we next examined whether cocaine exposure could also lead to alterations in excitatory and/or inhibitory synapses. For this, hippocampal neurons were exposed to cocaine as described above and the numbers of excitatory/inhibitory synapses were assessed by immunostaining using antibodies specific to the synaptic markers vGlut1 (excitatory) and vGAT (inhibitory). As shown in Figures 2a and b, cocaine exposure significantly increased the number of excitatory synapses (~52%) compared with the control cells. Interestingly, however, the number of inhibitory synapses did not change following cocaine exposure (Figures 2c and d). Balanced control of excitatory and inhibitory synapses is critical for maintaining normal brain functioning. When measured as the ratio of vGlut1 to vGAT puncta, it was clear that the balance of excitatory and inhibitory synapses was impaired in cocaine-exposed hippocampal neurons (Figure 2e). In addition, we determined the levels of excitatory synaptic markers, such as synaptophysin (presynaptic) and PSD95 (postsynaptic), in the lysates of cultured hippocampal neurons following exposure to cocaine. As shown in Figures 2f and g there was increased expression of synaptophysin (~55%) and PSD95 (~63%) in cocaine-treated hippocampal neurons compared with control cells. These findings demonstrate that cocaine selectively alters excitatory synapses in hippocampal neurons and suggest excitatory/inhibitory imbalance in neurotransmission as an underlying cause of cocaine-mediated cognitive dysfunction.

### *In vivo* effects of cocaine administration on spine density in the hippocampus and cerebral cortex

**Figure 3:**
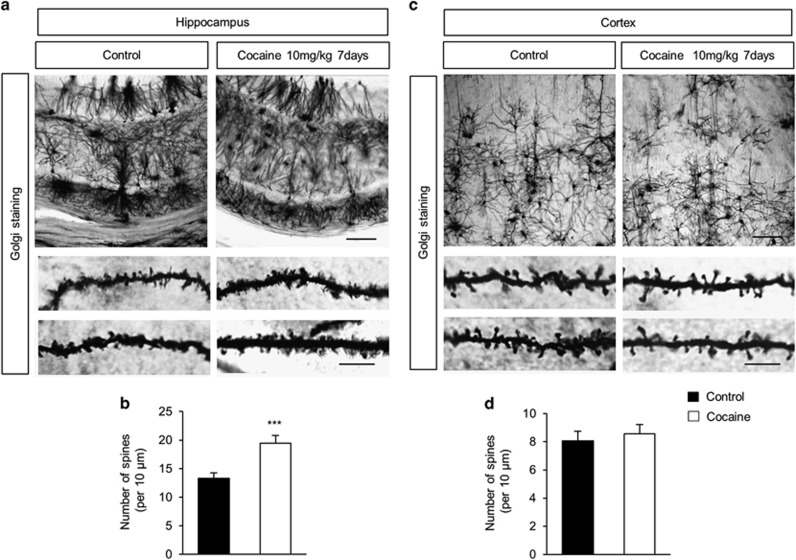
Cocaine administration changes the density of dendritic spines in the hippocampus. (**a**) Dendritic spines were assessed in hippocampal tissues from mice injected with either cocaine (10 mg/kg) or saline control intraperitoneally once a day for 7 consecutive days. Golgi staining showed an increase in dendritic spines in cocaine-treated samples. Scale bar, 50 *μ*m (top panels) and 10 *μ*m (bottom panels). (**b**) The number of dendritic spines was quantified. *n*=20 sections from three mice for each condition. Statistical significance was determined by two-tailed Student's *t*-test. ***P<0.001. (**c**) Cocaine administration did not change the spine density in cortical neurons *in vivo*. Scale bar, 50 *μ*m (top panels) and 10 *μ*m (bottom panels). (**d**) Quantification of (**c**). *n*=20 sections from three mice for each condition. Statistical significance was determined by two-tailed Student's *t*-test

Based on the differential effects of cocaine on spine density in cultured hippocampal *versus* cortical neurons, we sought to examine whether cocaine administration *in vivo* would have similar effects on dendritic spines. For this, adult mice were injected with either cocaine (10 mg/kg) or saline (control) intraperitoneally once a day for 7 consecutive days. Mice were then killed within 1 h of the last cocaine injection and brains were assessed for the number of dendritic spines by Golgi staining. Our findings revealed that cocaine administration resulted in the increased number of dendritic spines (~61%) in the hippocampus, compared with saline treated controls (Figures 3a and b). In contrast, cocaine administration did not alter the number of dendritic spines in the cerebral cortex (Figures 3c and d). These findings thus validate the differential effects of cocaine on spine density alteration in the hippocampus *versus* the cortex.

**Figure 4:**
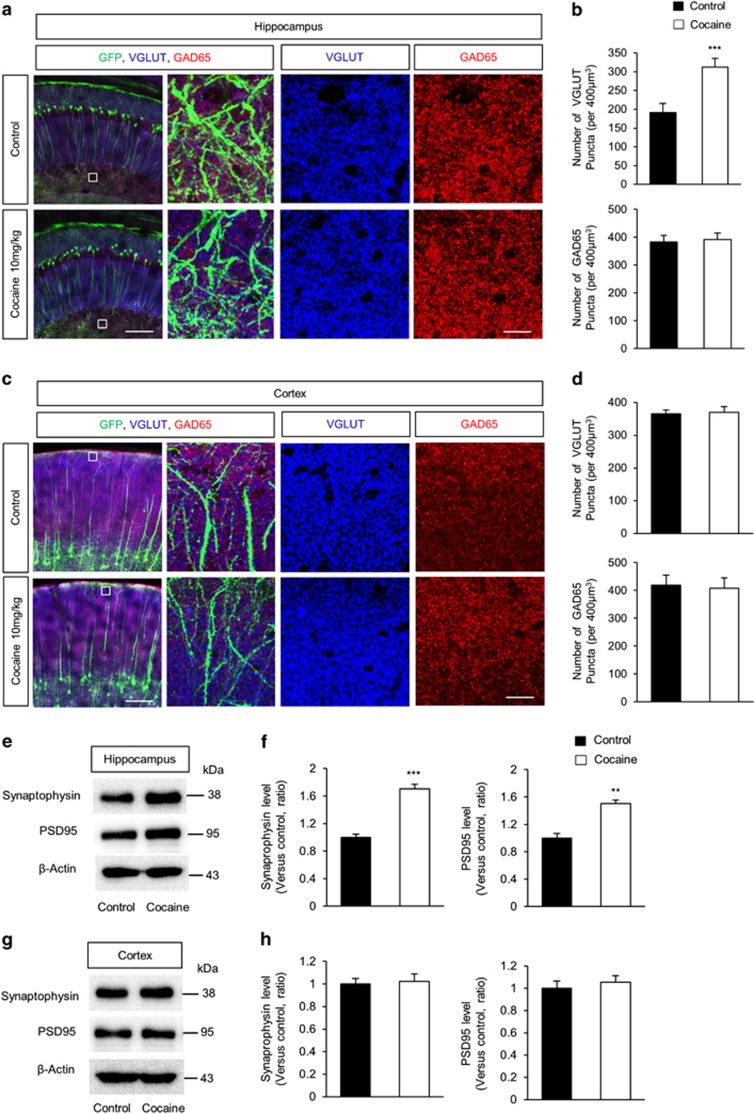
Cocaine administration increases excitatory synapses in the hippocampus. (**a**) Mice were injected with either cocaine (10 mg/kg) or saline (control) intraperitoneally once a day for 7 consecutive days. Then excitatory and inhibitory synapses were assessed by immunostaining with a vGlut1and GAD65 antibody. Scale bar, 50 *μ*m (left panels) and 20 *μ*m (right panels). Right three panels showed a higher magnification of the white square box in the left panel. (**b**) The numbers of synaptic puncta were quantified. *n*=20 sections from three mice for each condition. Statistical significance was determined by two-tailed Student's *t*-test. ***P<0.001. (**c**) Cocaine administration effect on cortical synapses *in vivo*. Scale bar, 50 *μ*m (left panels) and 20 *μ*m (right panels). (**d**) Quantification of synaptic puncta shown in (**c**). *n*=20 sections from three mice for each condition. Statistical significance was determined by two-tailed Student's *t*-test. (**e**) The levels of synaptic proteins were increased by cocaine administration in the hippocampus. After cocaine injection, western blotting was performed using a synaptophysin and a PSD95 antibody. (**f**) Quantification of protein levels shown in (**e**). The levels of protein were normalized to *β*-actin expression. *n*=3 independent brains using three mice. Statistical significance was determined by two-tailed Student's *t*-test. ***P<0.001. (**g**) The levels of synaptic proteins in the cortex were assessed after cocaine administration. (**h**) Quantification of (**g**). *n*=3 independent brains using three mice. Statistical significance was determined by two-tailed Student's *t*-test

We next assessed the numbers of excitatory and inhibitory synapses *in vivo* in the hippocampus and cerebral cortex of YFP mice administered either cocaine or saline. Following cocaine or saline exposure, hippocampi and cortices were immunostained with antibodies specific to vGlut1 (excitatory) and GAD65 (inhibitory). As shown in Figure 4a cocaine administration resulted in elevated numbers of vGlut1 puncta (~63%) in hippocampal neurons, compared with the saline control (Figure 4b). Similar to the cell culture findings, cocaine administration did not alter the numbers of vGlut1 or GAD65 puncta in cortical neurons (Figures 4c and d). We also measured the levels of synaptophysin and PSD95 using hippocampal and cortical lysates isolated from cocaine or saline-administered mouse brains. Cocaine administration resulted in increased levels of both synaptophysin (~55%) and PSD95 (~63%) proteins in the hippocampus with negligible changes in the cortex (Figures 4e–h).

### Cocaine regulates dendritic outgrowth in cultured hippocampal neurons

**Figure 5:**
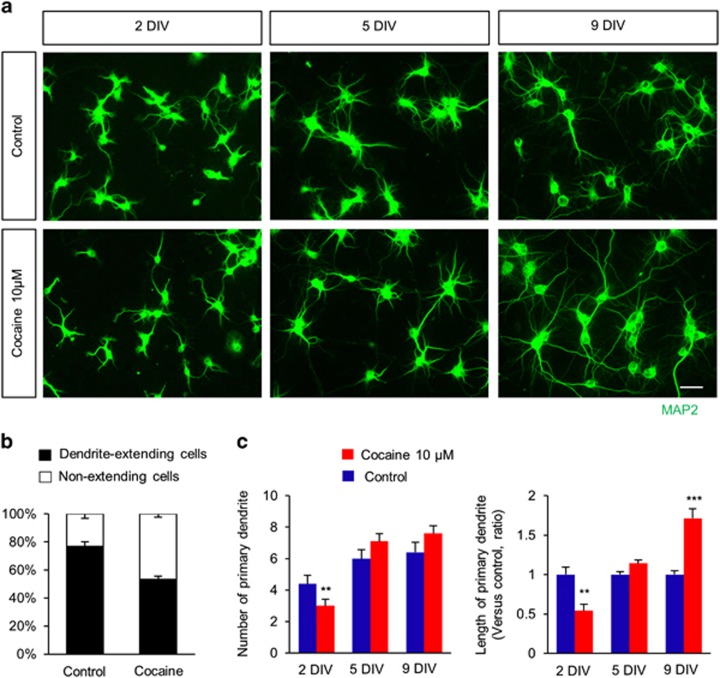
Cocaine effects on dendrite outgrowth. (**a**) Cocaine initially suppressed dendrite outgrowth, but promoted the outgrowth after 2 DIV in cultured hippocampal neurons. Cultured hippocampal neurons were treated with saline or cocaine (10 *μ*M) every other day for 2, 5, and 9 days. Dendrites were assessed by immunostaining using a MAP2 antibody. Scale bar, 10 *μ*m. (**b**) Quantification of the number of dendrite-extending neurons in each condition. *n*=1045 neurons for control; *n*=1192 neurons for the cocaine condition. (**c**) The numbers and lengths of dendrites were quantified. *n*=30 cells from three independent cultures using three rats for each condition. Statistical significance was determined by two-tailed Student's *t*-test. **P<0.01. ***P<0.001

As cocaine caused an increase in dendritic spines in hippocampal neurons, we examined whether cocaine also had a role in dendritic outgrowth in these cells. Hippocampal neurons were exposed to cocaine every other day for 9 days and were assessed for dendritic outgrowth by immunostaining with a MAP2 antibody. Cocaine exposure for 2 days decreased the number of dendrite-extending hippocampal cells by 30% compared with controls (Figures 5a–c). No significant differences in the dendrite number were found after 2 days. The length of primary dendrites was also decreased in 2-day cocaine-treated cells, but increased after 9 days by 71% compared with the length in controls (Figures 5a and c).

### Cocaine activates TrkB/BDNF signaling in hippocampal neurons

**Figure 6:**
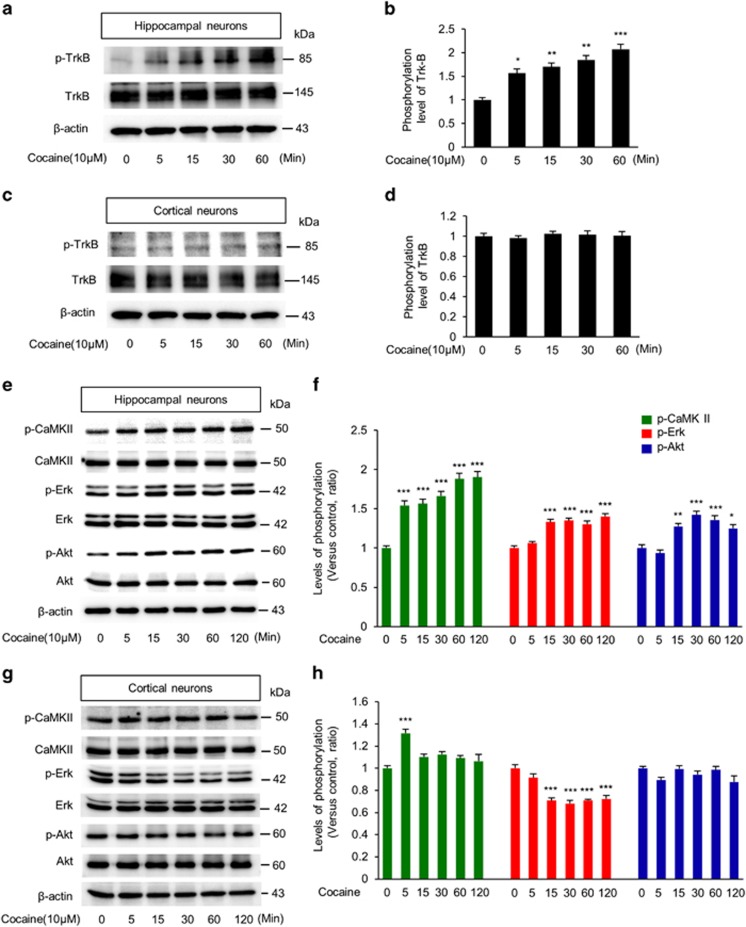

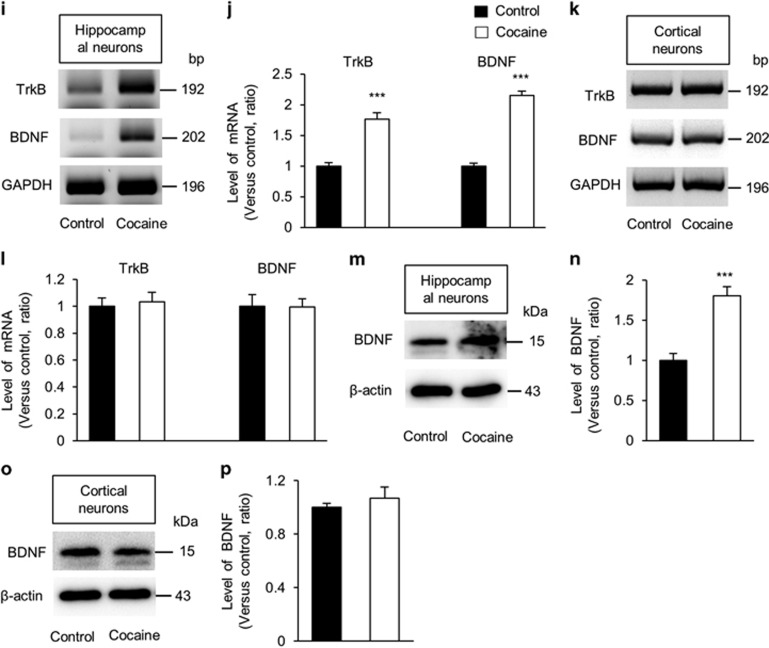
Cocaine activates TrkB signaling in hippocampal neurons. (**a**) Cocaine effects on TrkB activity in hippocampal cells was measured by western blotting. (**b**) Quantification of phosphorylated TrkB shown in (**a**). The level of phosphorylated TrkB was normalized to the total TrkB level. *n*=3 independent hippocampal cultures using three rats. Statistical significance was determined by two-tailed Student's *t*-test. **P<0.05, **P<0.01, ***P<0.001. (**c**) Cocaine did not activate TrkB in cortical neurons. (**d**) Quantification of (**c**). *n*=3 independent cortical cultures using three rats. (**e**) The levels of phospho-CaMKII, phospho-ERK, and phospho-AKT in hippocampal cells were measured after cocaine treatment. (**f**) Quantification of phospho-proteins shown in (**e**). The relative levels of phospho-proteins were normalized to total protein levels. *n*=3 independent hippocampal cultures using three rats. Statistical significance was determined by two-tailed Student's *t*-test. ** *P*<0.01. (**g**) The levels of phospho-CaMKII, phospho-ERK, and phospho-AKT in cortical cells were measured after cocaine treatment. (**h**) Quantification of (**g**). *n*=3 independent cortical cultures using three rats. Statistical significance was determined by two-tailed Student's *t*-test. *** *P*<0.001. (**I**) The mRNA levels of BDNF and TrkB were assessed by RT-PCR in hippocampal neurons treated with 10 * μ*M cocaine for 24 h. (**j**) Quantification of RT-PCR results in (**i**). The relative levels of the genes were normalized to the level of GAPDH mRNA. *n*=3 independent cultures using three rats. Statistical significance was determined by two-tailed Student's *t*-test. ***P<0.001. (**k**) The mRNA levels of BDNF and TrkB were assessed in cortical neurons after the cocaine treatment. (**l**) Quantification of (**k**). (**m**) The protein level of BDNF was examined in cocaine-treated hippocampal neurons by western blotting. (**n**) Quantification of (**m**). The relative level of the protein was normalized to *β*-actin expression. *n*=3 independent cultured hippocampal neurons using three rats. Statistical significance was determined by two-tailed Student's *t*-test. ***P<0.001. (**o**) The protein level of BDNF was examined in cocaine-treated cortical neurons by western blotting. (**p**) Quantification of (**o**)

To identify the signaling pathway involved in cocaine-induced changes in dendritic spines, we investigated BDNF/TrkB signaling that has been reported to have an important role in both dendrite and synapse formation.^38, 39, 40^ Hippocampal and cortical neurons were exposed to cocaine (10 *μ*M) for varying lengths of time, and their lysates were used to assess time-dependent expression of phospho-TrkB, a direct indicator of TrkB activity. As shown in Figures 6a and b, in cocaine-exposed hippocampal neurons, there was a time-dependent increase in the phosphorylation of TrkB. In contrast, cocaine exposure failed to impact the phosphorylation status of TrkB in cortical neurons (Figures 6c and d). The next step was to assess the activation of downstream mediators of the TrkB pathway in response to cocaine. Using western blotting we examined the phosphorylation levels of CaMKII, ERK, and AKT in hippocampal and cortical neurons following cocaine exposure. As shown in Figure 6e, in hippocampal neurons exposed to cocaine there was a time-dependent activation of CamKII, ERK, and AKT after cocaine exposure compared with non-treated control cells (Figure 6f). Contrary to hippocampal neurons, cortical neurons exhibited minimal changes in the phosphorylation levels of the downstream TrkB mediators in response to cocaine (Figures 6g and h). There was downregulation of ERK phosphorylation in cortical neurons following cocaine exposure. Taken together these findings suggest a role of TrkB and its downstream kinases in cocaine-induced spine alteration.

We also examined BDNF and TrkB mRNA and protein levels in hippocampal and cortical neurons exposed by cocaine. Total RNAs were isolated from neuronal cultures treated with cocaine for 24 h and subjected to RT-PCR. In the hippocampal neurons, cocaine exposure resulted in increased levels of TrkB and BDNF mRNAs compared with the control condition. In contrast, we found no change in BDNF or TrkB mRNA levels in cortical neurons exposed to cocaine (Figures 6i–l). Similar findings were observed in BDNF protein levels in response to cocaine in hippocampal and cortical neurons (Figures 6m–p).

Next we sought to assess the effect of cocaine on TrkB signaling *in vivo*. Three month-old mice were injected with either cocaine or saline intraperitoneally once per day for 7 consecutive days, followed by isolation of hippocampal and cortical lysates 1 h after the last cocaine injection. Similar to the findings in cell culture, cocaine administration resulted in increased BDNF and TrkB mRNA levels in the hippocampus compared with the saline control. There was no effect of cocaine in the cerebral cortex of same mice (Supplementary Figures 1a–d). In parallel, we also assessed the phosphorylation levels of TrkB and found it to be upregulated in the hippocampus of cocaine-administered mice (Supplementary Figures 1e and f), with no observable change in the cortex (Supplementary Figures 1g and h). Phosphorylation levels of TrkB downstream mediators, ERK and CaMKII were also increased in the cocaine-administered hippocampus compared with saline controls (Supplementary Figures 1i and j), with no change in the cortex (Supplementary Figures 1k and l). These results suggest that cocaine differentially regulates BDNF/TrkB signaling in the hippocampus and cortex.

### Participation of Sig-1R in cocaine-mediated TrkB activation and spine morphogenesis

**Figure 7:**
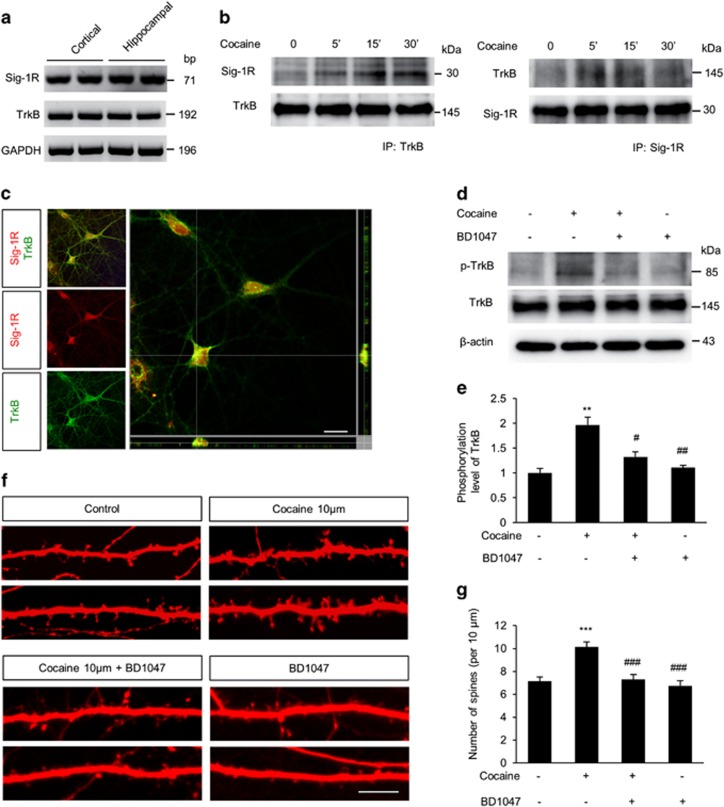
Sig-1 R is a key regulator in cocaine-induced TrkB activation and spine alteration. (**a**) Sig-1 R and TrkB were expressed in cultured hippocampal and cortical neurons. RT-PCR was performed using RNAs from E18 rat cortical and hippocampal lysates. (**b**) Cocaine promoted the interaction of Sig-1 R and TrkB. Cell lysates were co-immunoprecipitated with either a Sig-1 R or TrkB antibody, and subsequently subjected to western blotting using antibodies indicated in the figure. (**c**) Immunostaining and confocal microscopy showed that Sig-1 R colocalized with TrkB in cultured hippocampal neurons. Scale bar, 10* μ*m. (**d**) Western blotting showed that the treatment of cultured hippocampal neurons with Sig-1 R antagonist BD1047 (10 *μ*M) inhibited cocaine-induced activation of TrkB. (**e**) Quantification of phospho-TrkB shown in (**d**). The phospho-TrkB level was normalized to total TrkB. *n*=3 independent hippocampal cultures using three rats. Statistical significance was determined by two-tailed Student's *t*-test. **P<0.01 *versus* control group; ^#^*P*<0.05, ^##^*P*<0.01 *versus* cocaine-treated group. (**f**) The treatment with BD1047 suppressed the cocaine-mediated increase in dendritic spines. Scale bar, 10 *μ*m. (**g**) Quantification of spine numbers in each condition shown in (**f**). *n*=30 cells from five independent cultures using five rats for each condition. Statistical significance was determined by two-tailed Student's *t*-test. ***P<0.001 versus control group; ^###^*P*<0.001 *versus* cocaine-treated group

Cocaine is an agonist for Sig-1R, which interacts with receptor tyrosine kinases in cerebellar granule neurons.^18, 41, 42^ We hypothesized that cocaine-mediated alteration of hippocampal spine morphology could involve activation of both Sig-1 R and a receptor tyrosine kinase TrkB. Cultured hippocampal and cortical neurons expressed Sig-1 R as well as TrkB (Figure 7a). Next, we investigated whether cocaine exposure triggers a molecular interaction between Sig-1 R and TrkB in cultured hippocampal neurons. Co-immunoprecipitation assays revealed that cocaine exposure induced the interaction between Sig-1 R and TrkB (Figure 7b). TrkB is activated when it is phosphorylated, thus we also examined the interaction of Sig-1 R with phospho-TrkB. We found that the level of phospho-TrkB associated with Sig-1 R was increased after cocaine treatment in cultured hippocampal cells (Supplementary Figure 2), suggesting that Sig-1 R binding to TrkB induces TrkB activation. The Sig-1 R-immunoreactive band was seen in TrkB precipitates and reciprocally, the TrkB band was displayed in Sig-1 R precipitates. We also confirmed this interaction by immunostaining. Confocal z-stacked images showed TrkB and Sig-1 R were colocalized in the cell (Figure 7c), supporting an interactive nature for these molecules. These results suggest that Sig-1 R participates in the activation of TrkB signaling and subsequent dendritic spine alteration in cocaine-treated hippocampal neurons. Thus, we investigated whether Sig-1 R activates TrkB in hippocampal neurons after cocaine treatment. Hippocampal neurons were pretreated with a Sig-1 R antagonist, BD1047, for 1 h, followed by cocaine exposure for 15 min. Cellular lysates were subjected to western blotting for measuring phosphorylated TrkB levels. Pretreatment of neurons with BD1047 significantly inhibited cocaine-mediated activation of TrkB (Figures 7d and e), indicating Sig-1 R indeed mediates the cocaine effect on TrkB activation. Next, we examined if Sig-1 R is involved in cocaine-induced increase in spine numbers. At 10 days in-culture hippocampal neurons were transfected with a RFP-expressing plasmid. After 6 days, neurons were pretreated with the BD1047 for 1 h followed by incubation with cocaine for 24 h and then dendritic spines were assessed. As expected, the cocaine treatment increased the number of dendritic spines by 30% compared with the control (Figures 7f and g). Importantly, pretreatment of neurons with BD1047 suppressed the cocaine-induced increase in dendritic spines. These results suggest a critical role for Sig-1 R in TrkB signaling activation during cocaine-induced spine plasticity.

## Discussion

**Figure 8:**
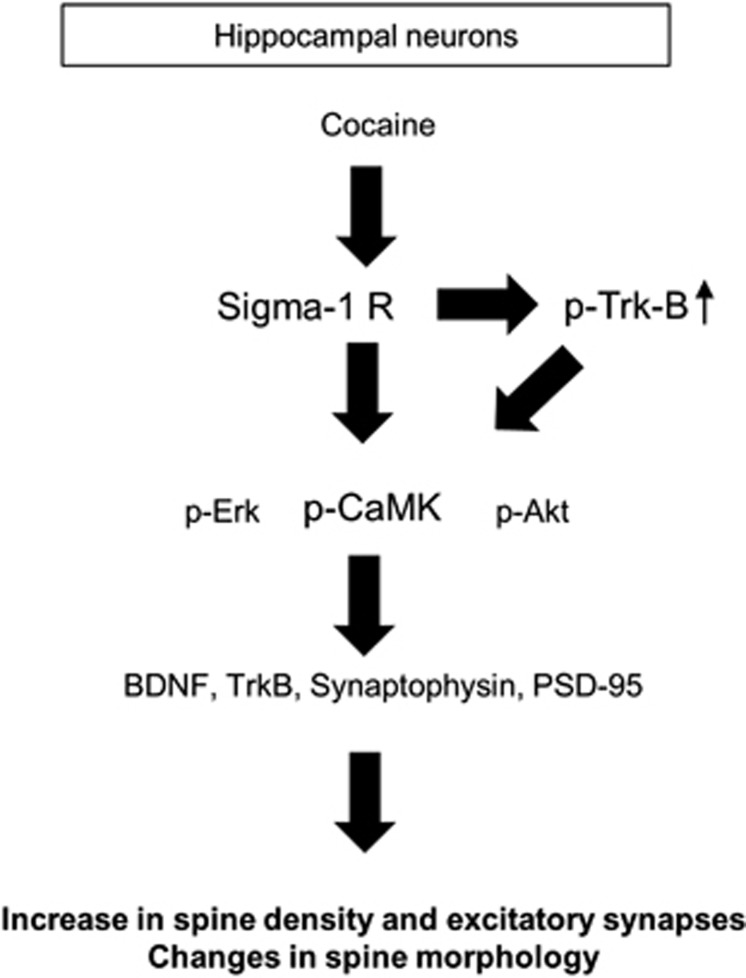
A schematic model illustrating an effect of cocaine on dendritic spine. Cocaine activates TrkB via Sig-1 R, leading to the activation of TrkB downstream kinases especially CaMKII in hippocampal neurons. Activation of cellular TrkB signaling induces gene expression of synaptic molecules and remodels dendritic spines

In this study, we provide evidence that cocaine differentially affects dendritic spine density and morphology in hippocampal *versus* cortical neurons via the regulation of TrkB signaling. In hippocampal neurons, transactivation of TrkB by Sig-1 R has an important role in cocaine-mediated dendritic spine impairment (Figure 8). Our results provide novel insights into anatomical and molecular targets for cocaine-mediated alterations in spine density. Understanding mechanisms of cell type-specific effects of cocaine could have implications for future development of therapeutic targets aimed at region-specific delivery to the brain.

We found that cocaine administration alters spine density and morphology, which is involved in the strength and turnover of synapses.^43, 44, 45, 46^ Cocaine exposure increases stubby spines and decreases thin spines in hippocampal neurons. Cocaine thus influences neural transmission by modifying pre-existing connections while also creating new contacts. The effect of cocaine on cortical neurons has been confounding owing to controversial results. For example, some studies have reported that cocaine exposure increases spines in cortical neurons,^43, 47^ whereas others have shown opposite effects in the same cells.^48, 49^ Our findings suggest no observable change in the spine density in cortical neurons exposed to cocaine. However, we did observe changes in spine morphology, with increased numbers of thin and fewer numbers of stubby and mushroom spines in cortical neurons following cocaine exposure. Alterations in neuronal plasticity induced by cocaine could thus involve a combination of factors comprised of cell specificity, spine morphology, and/or synaptic strength. Previous studies have shown that cocaine exposure enhances excitatory connectivity in the NA and the laterodorsal tegmental nucleus.^50, 51^ Consistently we have shown that cocaine increases the number of excitatory synapses in hippocampal cultures. This effect of cocaine was selective for excitatory synapses, as there was no significant change in the density of inhibitory synapses. Our findings suggest that cocaine administration may increase the content of NMDA receptor-rich glutamatergic neurons in the hippocampus. The changes in the number and composition of spines following cocaine exposure could have a role in synaptic alterations. These results thus suggest that addictive drugs, such as cocaine, could modify neuronal circuitry, especially addiction and reward circuits, by modifying the postsynaptic compartment of excitatory synapses. Whether presynaptic axon terminals are also affected by cocaine remains to be determined.

The abnormal spine density and morphology induced by cocaine might influence hippocampal dopamine signaling. Hippocampus receives dopaminergic innervations from the VTA.^18, 52, 53^ Dopamine signaling in the hippocampus has a critical role in addiction and reward pathways of the brain.^4, 17, 54, 55^ Cocaine has been shown to amplify dopamine signaling by increasing the availability of dopamine in the hippocampal synapses through inhibition of dopamine reuptake. It will be compelling to examine dopamine receptor levels in hippocampal neurons following cocaine treatment.

The molecular mechanisms underlying cocaine-induced neuroanatomical changes are not well understood. The role of BDNF/TrkB signaling in cocaine addiction has been documented. For example, cocaine induces BDNF expression^56, 57^ and activates TrkB in the NA.^58^ Overexpression of dominant-negative TrkB was shown to abrogate cocaine-induced behavioral sensitization.^59^ Detailed molecular signaling of TrkB activation, however, has remained unclear. Herein, we demonstrate that cocaine triggers Sig-1 R transactivation of TrkB in hippocampal neurons. This, in turn, leads to TrkB downstream activation of ERK, AKT, and CaMKII. We showed that short-term exposure (<1 h) with cocaine activated TrkB signaling by enhancing TrkB phosphorylation in hippocampal neurons. Within this time frame, the level of TrkB was not changed. However, the TrkB transcript level was increased after 24 h of cocaine treatment. Thus, cocaine appears to activate TrkB signaling by both genomic action and phosphorylation of TrkB. Pharmacological inhibition of Sig-1 R prevents activation of TrkB signaling and spine formation. Collectively, our results show that activation of TrkB signaling is a critical mechanism for differential role for cocaine in spine alterations. Our findings are in agreement with a report by Kimura *et al.*^41^ showing that the interaction of Sig-1 R with TrkB leads to neurite elongation in cerebellar granule neurons. Thus, therapeutic interventions aimed at specifically abrogating TrkB signaling in the hippocampal neurons could be considered as future options for treating cocaine addiction. A recent report has shown another pathway of Sig-1 R action in the cell.^60^ Cocaine as a Sig-1 R agonist dissociates Sig-1 R from its binding partner BiP in the endoplasmic reticulum and induces the translocation of Sig-1 R to the nuclear envelope. This translocation leads to cocaine-induced transcriptional regulation via the recruitment of chromatin remodeling molecules including barrier-to-autointegration factors and histone deacetylases.^60^ In addition to transactivation of TrkB, this pathway may also contribute to the cocaine-induced alteration in dendritic spines by changing the levels of gene transcripts associated with spine density and morphology.

## Materials and Methods

### Plasmid

pSuper-venus vector was described previously.^61^ pAAV-CAG-tdTomato was purchased from Addgene (Cambridge, MA, USA).

### Animals

Mice (C57BL/6 N) and rats (Sprague Dawley) were handled according to our animal protocols approved by the University of Nebraska Medical Center. YFP-H mouse (strain B6.Cg-Tg (thy-1-YFPH) 2Jrs/J) was obtained from The Jackson Laboratory (Bar Harbor, ME, USA).

### Primary neuronal cultures

Primary neuronal culture was described previously.^62, 63^ In brief, cerebral cortices from E18 rats were isolated and dissociated with trituration after trypsin/EDTA treatment. Then, the cells were plated onto poly-D-lysine/ laminin-coated coverslips and cultured in the medium containing neurobasal medium, 5% serum, B27 and N2 supplements.

### Cell transfection

Neuronal transfection was performed as described in previous papers.^64, 65^ DNA constructs were transfected into attached cells using lipofectamine (Thermo Fisher Scientific Inc., Waltham, MA, USA) according to the manufacturer's protocol.

### Immunostaining

Immunostaining of brain sections and dissociated cells were performed as described previously.^66, 67^ The following primary antibodies were used: Chicken anti-GFP (Thermo Fisher Scientific Inc.), rabbit anti-GFP (Thermo Fisher Scientific Inc.), rabbit anti-RFP (Thermo Fisher Scientific Inc.), mouse anti-MAP2 (BioLegend, San Diego, CA, USA), guinea pig anti-vGlut (EMD Millipore, Billerica, MA, USA), rabbit anti-vGAT (PhosphoSolutions, Aurora, CO, USA), mouse anti-GAD6 (DSHB) and mouse anti-Sig-1 R (Santa Cruz Biotechnology, Inc., Dallas, TX, USA)). Appropriate secondary antibodies conjugated with Alexa Fluor dyes (Thermo Fisher Scientific Inc) were used to detect primary antibodies.

### Morphometry

For the quantification of dendritic spines in cultured neurons, images from five independent cultures using five rats were taken with Zeiss LSM510 and LSM710 confocal microscopes. Cell numbers examined were described in figure legends. For the quantification of length or number of dendrite, images of three independent cultures using three rats were taken Nikon Eclipse epifluorescence microscope attached with a Q Imaging CCD camera. Cell numbers examined were described in figure legends. The images were analyzed by using ZEN (Carl Zeiss, Oberkochen, Germany), LSM image browser (Carl Zeiss), QCapture software (Q Imaging, Surrey, BC, Canada), and ImageJ (NIH, Bethesda, MD, USA). The calculated values were averaged, and some results were recalculated as relative changes *versus* control. All counting and classification of dendritic spines was carried out by one trained individual. Synapses in mouse brains, images of 20 different brain sections from more than three mice for each condition were used for confocal microscopy. The number of synapses was assessed by immunostaining the cultures or brain sections with antibodies to excitatory (vGlut1) and inhibitory (GAD65) synaptic markers, and by counting vGlut or GAD65 puncta. Images were processed by Imaris software (Bitplane AG, Zurich, Switzerland).

### Golgi staining

Golgi-Cox staining to obtain hippocampus and cortex spine density was conducted with the FD Rapid GolgiStain Kit (FD Neuro Technologies, Inc., Columbia, MD, USA) according to the manufacturer's instructions. Coronal tissue sections of 180-*μ*m thicknesses were made at room temperature using the VT1000S (Leica Biosystems Inc., Buffalo Grove, IL, USA). Sections were dehydrated with a gradient of 50, 75, 95 to 100% ethanol and cleared in xylene.

### Western blotting

Western blotting was performed as described previously.^68, 69^ Lysates from brains or cultured cells were prepared using RIPA buffer and the protein content was determined by a Bio-Rad Protein Assay system. Proteins were separated on 4–12% SDS-PAGE gradient gel and transferred onto nitrocellulose membrane. Then the membrane was incubated with mouse anti-β-Actin (Sigma-Aldrich, Inc., St. Louis, MO, USA), rabbit anti-BDNF (Abcam, Cambridge, MA, USA), rabbit anti-TrkB (Abcam), rabbit anti-p-TrkB (Abcam), rabbit anti-CaMKII (Santa Cruz Biotechnology, Inc.), mouse anti-p-CaMKII (Santa Cruz Biotechnology, Inc.), rabbit anti-AKT (Cell Signaling Technology, Inc., Danvers, MA, USA), rabbit anti-p-AKT (Cell Signaling Technology, Inc.), mouse anti-ERK (Cell Signaling Technology, Inc.), rabbit anti-p-ERK (Cell Signaling Technology, Inc.), rabbit anti-synaptophysin (Cell Signaling Technology, Inc.), rabbit anti-PSD95 (Cell Signaling Technology, Inc.), mouse anti-Sig-1 R (Santa Cruz Biotechnology, Inc.) and mouse anti-Sig-1 R (Proteintech Group, Inc., Rosemont, IL, USA) at 4 °C overnight. Appropriate secondary antibodies conjugated to HRP were used (Cell Signaling Technology, Inc.) and the ECL reagents (GE Healthcare Bio-Sciences, Pittsburgh, PA, USA) were used for immunodetection. For quantification of band intensity, blots from three independent experiments for each molecule of interest were used. Signals were measured using ImageJ software and represented as relative intensity *versus* control. *β*-Actin was used as an internal control to normalize band intensity.

### Immunoprecipitation

Cellular lysates from cultured neurons treated with saline or cocaine (10 *μ*M) were prepared by using the Mammalian Cell Lysis kit (Sigma-Aldrich Inc.). For each sample, 600 *μ*g of protein was used for immunoprecipitation. Cell lysates were incubated with a Sig-1 R or TrkB antibody overnight at 4 °C followed by incubation with 30 *μ*l of protein A/G beads (Santa Cruz Biotechnology, Inc.) for 1.5 h at 4 °C. The mixture was then centrifuged at 12 000 rpm for 1 min, and the cell pellets were rinsed twice with the lysis buffer containing 1.0% NP-40, 0.5% sodium deoxycholate, 0.1% SDS, 150 mM NaCl, 9.1 mM Na_2_HPO_4_, 1.7 mM NaH_2_PO_4,_ 1% proteinase inhibitor, and 1% phosphatase inhibitor cocktail (Sigma-Aldrich Inc.). Finally, the cell pellets were subjected to Western blotting using a Sig-1 R antibody or TrkB antibody.

### Reverse transcription RCR

RNA was extracted from cultured neurons using TRIZOL reagent (Thermo Fisher Scientific Inc.), and cDNA was synthesized from 1 *μ*g of total RNA using oligo-dT and random hexamers using the Verso cDNA synthesis kit (Thermo Fisher Scientific Inc.). PCR was performed using 1 *μ*l of cDNA and the Master Mix (Promega Corporation, Madison, WI, USA). The sequences of the primers used were BDNF forward 5′-GCGGCAGATAAAAAGACTGC-3′ and reverse 5′-CCCGAACATACGATTGGGTA-3′, TrkB forward 5′-TGGTGCATTCCATTCACTGT-3′ and reverse 5′-CTTGGCCATCAGGGTGTAGT-3′, GAPDH forward 5′-AAGGTCATCCCAG AGCTGAA-3′ and reverse 5′-AGGAGACAACCTGGTCCTCA-3′.

### Statistical analysis

Normal distribution was tested using Kolmogorov–Smirnov test and variance was compared. Unless otherwise stated, statistical significance was determined by two-tailed Student's *t*-test for two-population comparison and one way analysis of variance followed by Bonferonni correction test for multiple comparisons. Data were analyzed using GraphPad Prism (GraphPad Software, Inc., La Jolla, CA, USA) and presented as mean (+/−) S.E.M. *P*-values were indicated in figure legends.

## Abbreviations

TrkB: tropomyosin receptor kinase B
Sig-1 R: sigma-1 receptor
NA: nucleus accumbens
VTA: ventral tegmental area
BDNF: brain-derived neurotrophic factor
ERK: extracellular signal-regulated kinase
PLC-γ: phosphoinositide phospholipase C-gamma
AKT/PKB: protein kinase B
CaMKII: Ca^2+^/calmodulin-dependent kinase II
CREB: cAMP response element-binding protein
GTP: guanosine-5'-triphosphate
E18: embryonic day 18
vGlut1: vesicular glutamate transporter 1
vGAT: vesicular GABA transporter
PSD95: postsynaptic density protein 95
GAD65: glutamic acid decarboxylase 65
MAP2: microtubule-associated protein 2
NMDA: N-methyl-d-aspartate
GFP: green fluorescent protein
RFP: red fluorescent protein
BiP: binding immunoglobulin protein
DIV: days *in vitro*
