# Supplementary material for: Transactivation of TrkB by Sigma-1 receptor mediates cocaine-induced changes in dendritic spine density and morphology in hippocampal and cortical neurons
Source: Cell Death Dis. 2016 Oct 13;7(10):e2414–. doi: 10.1038/cddis.2016.319 (PMC5133986; doi:10.1038/cddis.2016.319)
Supplement: Supplementary Figure Legends [file cddis2016319x1.docx]

**Supplementary Figure 1. Cocaine regulates TrkB signaling in the hippocampus**

(a) Cocaine increases BDNF and TrkB mRNA in the hippocampus. Three month-old mice were injected with saline or cocaine (10 mg/kg) intraperitoneally for seven consecutive days. Tissue lysates from the hippocampus were subjected to RNA isolation and RT-PCR. (b) Quantification of (a). The relative levels of the genes were normalized to GAPDH expression. n = 3 independent brains using 3 mice. Statistical significance was determined by two-tailed Student’s t-test. ***p*< 0.01. (c) Measurement of BDNF and TrkB mRNA levels in the cortex. (d) Quantification of (c). n = 3 independent brains using 3 mice. (e) Western blotting using a phospho-TrkB antibody showed that cocaine administration induced TrkB activation in the hippocampus. (f) Quantification of the phospho-TrkB level in (e). n = 3 independent brains using 3 mice. Statistical significance was determined by two-tailed Student’s t-test. ***p*< 0.01. (g) Cocaine did not alter the phospho-TrkB level in the cortex. (h) Quantification of (g). n = 3 independent brains using 3 mice. (i) Phosphorylation levels of ERK and CamKII were measured in the hippocampus of cocaine-treated mice using Western blotting. (j) Quantification of (i). n = 3 independent brains using 3 mice. Statistical significance was determined by two-tailed Student’s t-test. **p*< 0.05, ***p*< 0.01. (k) Phosphorylation levels of ERK and CamKII were measured in the cortex of cocaine-treated mice. (l) Quantification of (k). n = 3 independent brains using 3 mice.

**Supplementary Figure2. Cocaine increases the level of phospho-TrkB binding to Sig-1R**

Cellular lysates from cultured hippocampal neurons were immunoprecipitated with either a Sig-1R or p-TrkB antibody, and subsequently subjected to Western blotting using indicated antibodies.
