# Supplementary figures and images for: Transactivation of TrkB by Sigma-1 receptor mediates cocaine-induced changes in dendritic spine density and morphology in hippocampal and cortical neurons
Source: Cell Death Dis. 2016 Oct 13;7(10):e2414–. doi: 10.1038/cddis.2016.319 (PMC5133986; doi:10.1038/cddis.2016.319)

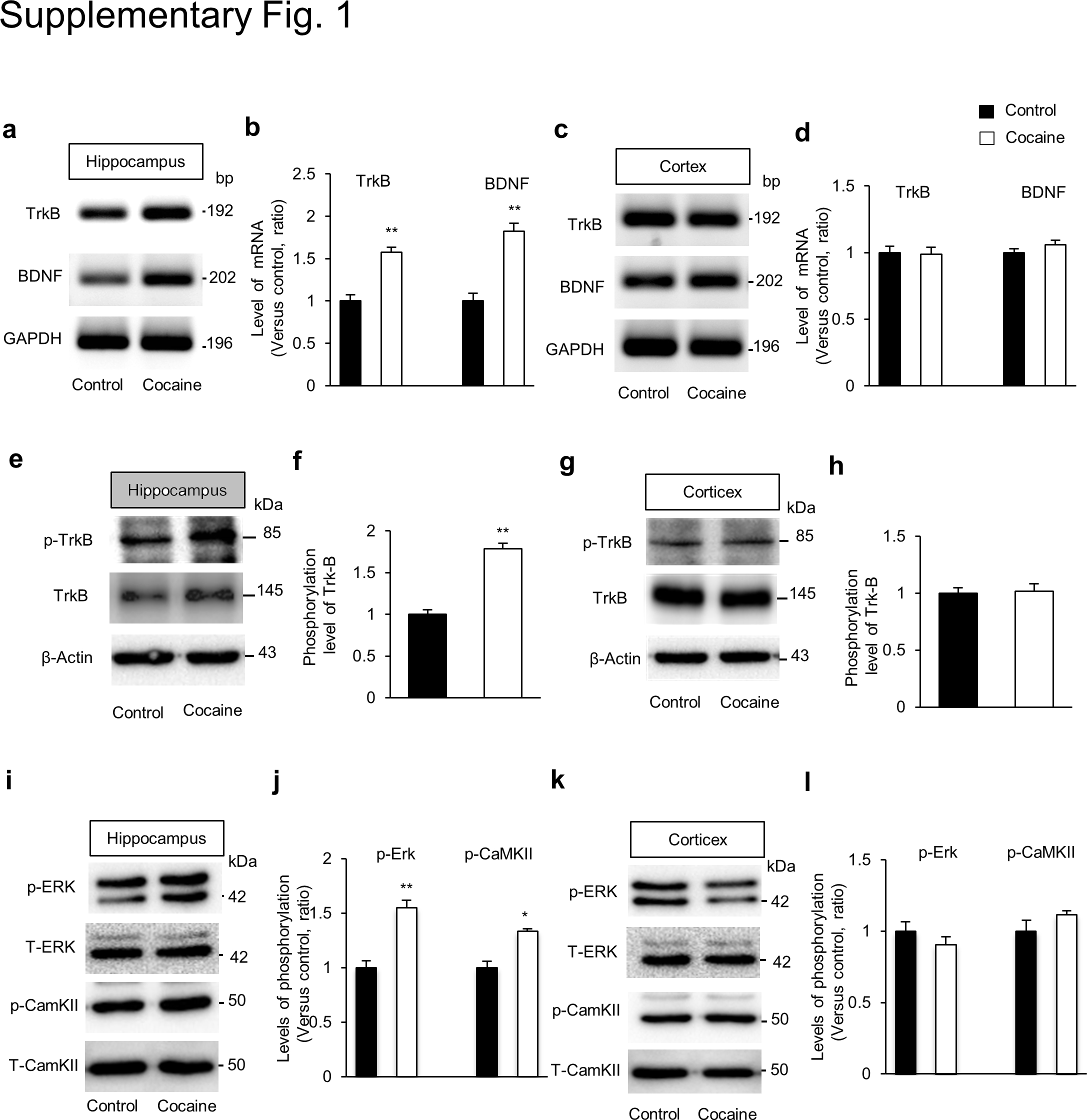

Supplement: Supplementary Figure 1 [file cddis2016319x2.tif]

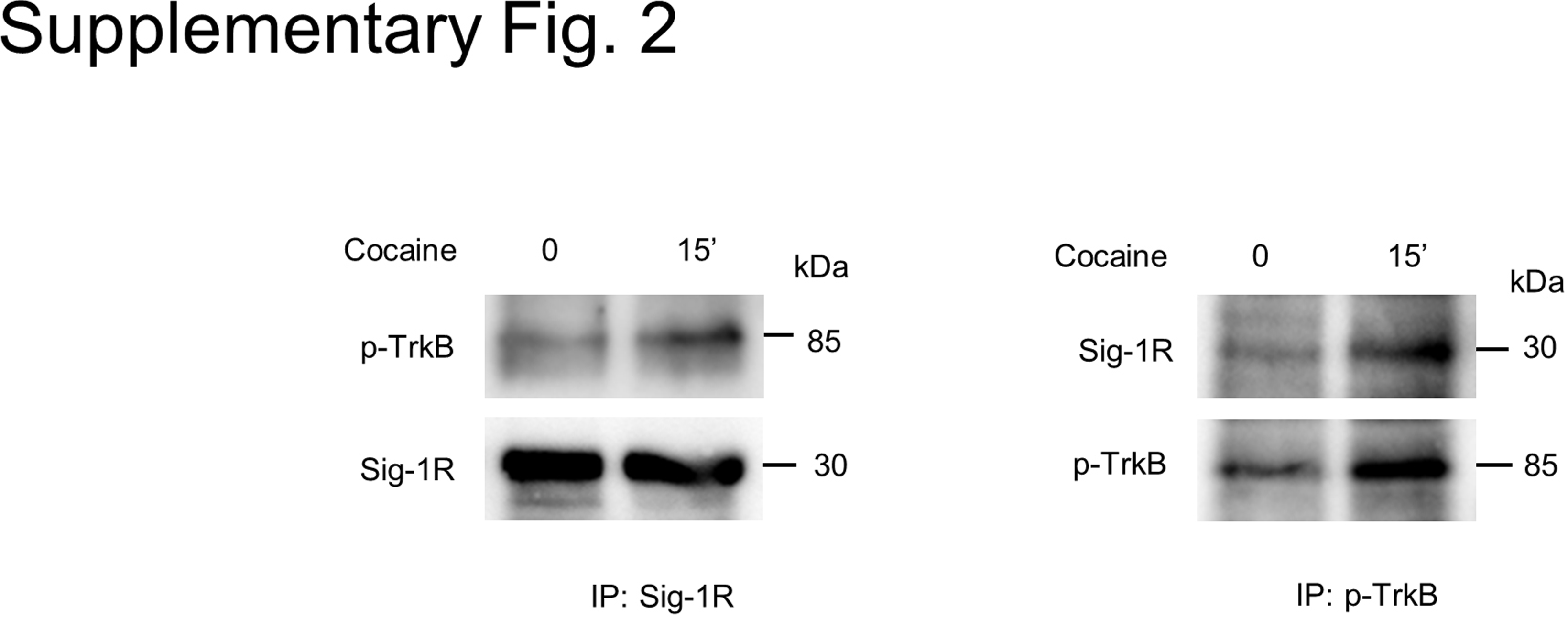

Supplement: Supplementary Figure 2 [file cddis2016319x3.tif]
